# Supplementary material for: Patterns of Intron Gain and Loss in Fungi
Source: PLoS Biol. 2004 Nov 30;2(12):e422. doi: 10.1371/journal.pbio.0020422 (PMC532390; doi:10.1371/journal.pbio.0020422)
Supplement: Table S1 — Also available at http://genes.mit.edu/NielsenEtAl/. (4.3 MB ZIP). [file pbio.0020422.st001.zip › NielsenEtAl/html/460.html]

AN1150.1.NCU05410.1.MG02375.1.FG01573.1


```
 CLUSTAL W (1.82) Multiple Sequence Alignments - Introns Inserted


Sequence 1: NCU05410.1	461 aa
Sequence 2: MG02375.1	567 aa
Sequence 3: FG01573.1	446 aa
Sequence 4: AN1150.1	468 aa
Alignment Length: 580 aa
Number Identitical Residues: 220 aa
Alignment Score (without introns) 10863


MG02375.1 	MAFRFVAHRIPANLALRSGSPIRFYSQAAAAFAAKANETQ0QASK~--LSNPDPAEDSAS
NCU05410.1	MAFRTALRRVAA---VNAAPATRLAGAGAGAATAARRSYA~TASQ~--LTHPDPTEDSPS
FG01573.1 	MAMRLPLR-------------ARLASS--CAIARPRIVRR~GLAS~--LPNADVASDSKS
AN1150.1  	MSFVRAASR-------RMPLAKRLVSAVESPMQRRYVSAT~LKAQ0GAVVTPDPGADSVS
          	*::                 . *: .    .            :. .::  .*   ** *

MG02375.1 	AALVREHAPFMVATYSRPSPVFVRAKGSYLWDMEERRYLDFTAGIAVTGLGHCDPQVSDI
NCU05410.1	GKMVREHVPYMVTTYSRPPPVFVKGKGSYLWDLEDRKYLDFTSGIAVNSLGHCDEEFSKI
FG01573.1 	AAMVNEHAPYMVATYARPPPVFIKGKGSWLWDAEDRKFLDFTAGIAVNGLGHCDPEFTRI
AN1150.1  	AAFVNERAPYLVPTYVRPAPIMVKGQGCYLWDMENRRYLDLTAGIAVNSLGHCDPEISKI
          	. :*.*:.*::*.** **.*::::.:*.:*** *:*::**:*:****..***** :.: *

MG02375.1 	LAEQ~GSTLMHTSNLYYNPWTLALSKALVEKTVASGGMHDAKSVFVCNSGSEANEAAIKF
NCU05410.1	IAEQ0AQELVHASNLYYNPWTGALSKLLVESTKASGGMHDASSVFVCNSGSEANEAGIKF
FG01573.1 	MTEQ0AQTLVHASNLYYNPWTGALSKLLVEKTLESGAMHNAASVFVCNSGSEANEAAIKF
AN1150.1  	IAEQ0AETLLHASNLYHNAWVGALSKDLIILTRESGAMRDAAQVFIANSGTEANEAAIKF
          	::** .. *:*:****:*.*. **** *:  *  **.*::* .**:.***:*****.***

MG02375.1 	ARKVGKVVDPSGNKTEIVSFANAFHGRTMGSLSATHNPKYQEPFAPMVPGFKCGTYNDIA
NCU05410.1	ARKVGKVLDPSGSKVEIVCFQNAFHGRTMGSLSATPNPKYQAPFAPMVPGFKVGTYNDIA
FG01573.1 	ARKAGKIVDPSGEKVEIVSFNNGFHGRTMGSLSATPNPKYQKPFSPMVPGFKTGDYNDIA
AN1150.1  	ARKTGRARDPSGAKHEIVSFHNSFHGRTMGALSATPNPKYQTPFSPMLPGFKYGKYNDVA
          	***.*:  **** * ***.* *.*******:**** ***** **:**:**** * ***:*

MG02375.1 	AIPDLVTEKTCGVIVEPIQGEGGVQVATDDFMVALAKRCREVGALLIHDEIQCGMARTGS
NCU05410.1	AIPSLVTEKTCSVIVEPIQGEGGVMPATEEFLVALGKRCREVGALLHYDEIQCGLARTGT
FG01573.1 	GIDALVTEKTCGVIVEPIQGEGGVTPAKEEFLVALAKRCREVGAVLIYDEIQCGLGRTGT
AN1150.1  	QLKDLVTEKTCGVIVEPIQGEGGVNVATPEFLSALRKRCDDVGAVLIFDEIQCGLSRTGS
          	 :  *******.************  *. :*: ** *** :***:* .******:.***:

MG02375.1 	LWAHGR---LP---KEAHPDMVTTAKALGNGFPIGAIIVN-GHVTEKIKVGDHGTTYGGN
NCU05410.1	FWAHSS---LP---KEAHPDILTTAKAIGNGFPIAATIVN-EHVASKIKVGDHGTTFGGN
FG01573.1 	FWAHGN---LP---KEAHPDILTTAKALGNGFPIGAVLVT-QDVADKMKVGDHGTTFGGN
AN1150.1  	FWAHAHPSLVPSSGEAAHPDILTTAKALGNGIPIGATIVSGKTVADNIKAGDHGTTFGGN
          	:***. .: :*::.: ****::*****:***:**.* :*..  *:.::*.******:***

MG02375.1 	PLACRLAHHVLERLSNPKLHESVMGKGAVLARGLAQLRQKHSELISEIRGRGLILGLQLT
NCU05410.1	PLACRLAHYIVGRLADKQLQEGVKAKSEVFLRGFEKLRNKFPSLVKEVRGKGLILGLQLS
FG01573.1 	PLACRLAHYIVGRLADKQLQADVKAKSEIFKKRFAKLQSQYPDLVKEVRGRGLILGLQLS
AN1150.1  	PLACRVAHHIIHRLASPELQKSVQDKSAILVAGFQALQQKYPDLVSEVRGRGLILGLQLS
          	*****:**::: **:. :*: .*  *. ::   :  *:.:...*:.*:**:********:

MG02375.1 	QDPAP----IVAAARERGLLIITAGHNTIS2NAARKKAG1DARAGEVALVPAERAVGNTA
NCU05410.1	EDPTP----VIKAARERGLLVITAGTNTLR~--------~--------FVPSLLVTEG--
FG01573.1 	QDPSP----IIKAARERGLLIITAGTNTLR~--------~--------FVPSLTVTDE--
AN1150.1  	KDFSARAGEIVTAARERGMLIITAGEGCLR~--------~--------FVPPLTITEE--
          	:* :. :..:: ******:*:**** . :                   :**.   .    

MG02375.1 	WEVRWPWAAPTAVEYLEQRSLTAGAPVGFYENQEGRQGQNSSRCLAGTINPSWLPSSHRD
NCU05410.1	------------------------------EIEEG-------------------------
FG01573.1 	------------------------------EINQG-------------------------
AN1150.1  	------------------------------QLKTA-------------------------
          	                              : : .                         

MG02375.1 	RISRWAAYDRYASILAEELAVRRVGRYASGDYVKAEVKTLTQKRY
NCU05410.1	-----------LKILEESFEAVMVKA-------------------
FG01573.1 	-----------LDIVETAIAATQ----------------------
AN1150.1  	-----------LRILEQAFEAVKA---------------------
          	             *:   : .
```
